# Rhamnetin ameliorates non-alcoholic steatosis and hepatocellular carcinoma in vitro

**DOI:** 10.1007/s11010-022-04619-6

**Published:** 2022-12-10

**Authors:** Mahmoud A. Shatta, Marwa O. El-Derany, Abdullah A. Gibriel, Hala O. El-Mesallamy

**Affiliations:** 1 Department of Biochemistry, Faculty of Pharmacy, The British University in Egypt, Cairo, 11837 Egypt; 2 Department of Biochemistry, Faculty of Pharmacy, Ain Shams University, Cairo, 11566 Egypt; 3 Dean of Faculty of Pharmacy, Sinai University, North Sinai, 45518 Egypt

**Keywords:** Rhamnetin, Fatty liver, Oleic acid, Palmitic acid, HepG2, Hepatocellular carcinoma

## Abstract

Non-alcoholic fatty liver (NAFLD) is a widespread disease with various complications including Non-alcoholic steatohepatitis (NASH) that could lead to cirrhosis and ultimately hepatocellular carcinoma (HCC). Up till now there is no FDA approved drug for treatment of NAFLD. Flavonoids such as Rhamnetin (Rhm) have been ascribed effective anti-inflammatory and anti-oxidative properties. Thus, Rhm as a potent flavonoid could target multiple pathological cascades causing NAFLD to prevent its progression into HCC. NAFLD is a multifactorial disease and its pathophysiology is complex and is currently challenged by the ‘Multiple-hit hypothesis’ that includes wider range of comorbidities rather than previously established theory of ‘Two-hit hypothesis’. Herein, we aimed at establishing reliable in vitro NASH models using different mixtures of variable ratios and concentrations of oleic acid (OA) and palmitic acid (PA) combinations using HepG2 cell lines. Moreover, we compared those models in the context of oil red staining, triglyceride levels and their altered downstream molecular signatures for genes involved in de novo lipogenesis, inflammation, oxidative stress and apoptotic machineries as well. Lastly, the effect of Rhm on NASH and HCC models was deeply investigated. Over the 10 NASH models tested, PA 500 µM concentration was the best model to mimic the molecular events of steatosis induced NAFLD. Rhm successfully ameliorated the dysregulated molecular events caused by the PA-induced NASH. Additionally, Rhm regulated inflammatory and oxidative machinery in the HepG2 cancerous cell lines. In conclusion, PA 500 µM concentration is considered an effective in vitro model to mimic NASH. Rhm could be used as a promising therapeutic modality against both NASH and HCC pathogenesis.

## Introduction

Non-alcoholic fatty liver disease (NAFLD) is a non-communicable disease affecting the liver without alcohol incorporation, with comorbidities ranging from steatosis to cirrhosis [1]. Several major liability factors are associated with NAFLD development including age, gender, obesity, diabetes mellitus type 2 (T2DM), hypertension, hyperlipidemia, unhealthy lifestyle, genetic and epigenetic variations [2, 3]. NAFLD is now one of the leading causes of hepatocellular carcinoma (HCC) that knocked over other contributing factors such as viral and alcohol-related liver diseases [4, 5]

In fact, NAFLD is a complicated disease with indefinite pathophysiology and involves lipids accumulation, peroxidation, inflammation, and oxidative stress [6–8]. Several proposals attempted to explain the cascade of NAFLD events. The “Two-Hit Hypothesis” was one of the major proposals, however, this theory is now considered obsolete as the “Multiple-Hit Hypothesis” is considered more reliable and representative. The “Multiple-Hit Hypothesis” is associated with the genetic and epigenetic dispositions, increased de-novo lipogenesis that affects triglycerides pool, gut dysbiosis as the flora increases fatty acids accumulation through increased production and uptake resulting in over production of proinflammatory cytokines, mitochondrial dysfunction, and fibrogenesis consequences that may develop cirrhosis or ultimately hepatocellular carcinoma (HCC) [9–11].

NAFLD is characterized by excessive inflammatory processes that if persist would lead to the development of Non-alcoholic steatohepatitis (NASH) which is an aggressive form of the disease as it causes steatosis and liver injury. As a natural compensatory mechanism, the liver would attempt initially to heal this injury and wound through the formation of fibrotic tissues of extracellular collagen matrix. Persistence of fibrosis would progress into cirrhosis which is an irreversible process with formation of hepatic scars that could ultimately develop HCC [12–15]. The latter is a life-threatening NAFLD complication that is considered the fourth cancer type to cause death worldwide

Although NAFLD was extensively studied in many situations, it is considered a perplexing silent disease without precise diagnosis except upon biopsy or appearance of severe fibrotic signs which are not usually consistent [14, 15]. This is due to incomplete understanding of the pathophysiology of this disease [16].

Kyoto encyclopedia of genes and genomes (KEGG) announced a genetic map for NAFLD/NASH (https://www.genome.jp/pathway/hsa04932) [17–19]. These maps and the reported molecular markers in literature aided in quoting related molecular targets to be investigated in our research. The molecular markers included in this project were sterol regulatory element binding protein (SREBP-1c) and fatty acid synthase (FAS) for investigating de-novo lipogenesis, glutathione reductase (GR), and catalase (CAT) for determination of oxidative stress, nuclear factor kappa-B (NFκB), interleukin 6 (IL-6), nuclear factor erythroid 2 (NFE-2), and tumor necrosis factor-alpha (TNF-α) for measuring the level of inflammation, tumor growth factor-beta (TGF-β) for fibrosis, and B-cell lymphoma 2 (BCL-2) for apoptosis.

To the best of our knowledge, no pharmacological treatment has yet been approved by the FDA neither NAFLD nor for NASH complications [20]. Flavonoids are natural compounds that were reported to possess various biological activities and to be effective as therapeutic remedies against several diseases [24]. Rhamnetin (Rhm) is a potent flavonoid with various effects reported, but it had not been investigated before against NAFLD/NASH or HCC disorders. The strong anti-oxidative activity of Rhm was reported to protect cardiac tissues from hydrogen peroxide toxic activity in vitro [21] as well as being an effective anti-inflammatory agent in vivo [22]. Furthermore, Rhm anti-inflammatory effect minimized some pro-inflammatory cytokines such as tumor necrosis factor (TNF) [23]. Another important finding was that Rhm induced sensitization of chemotherapeutic agents against HCC in vitro cell lines [24]. Recently Rhm antioxidative activity against SOD and CAT was confirmed in vivo in Ehrlich induced tumor in mice [25]. Moreover, Rhm showed apoptotic effect against breast cancer cell lines [26], and it was proven to enhance sorafenib activity against HCC by decreasing its metabolic clearance; but was not tested before against HCC alone [27]. The previous data supports the suggestion that Rhm might aid in amelioration of NAFLD and HCC because of its multi-faceted properties.

Thus, understanding the molecular mechanisms underlying NAFLD complications and its progression to NASH using a non-invasive technique is very crucial to determine possible and effective drugs. Unfortunately, this is hampered by the availability of suitable cellular models. On the other side, the long-term *in vivo* model setting and preparations that may extend to months with limited reproducibility, high interspecies variability in animals, high screening cost or legislative restrictions are all serious challenges that favor setting in vitro experiments especially at the beginning of any research to save time, cost and effort [28]. Accordingly, this study aimed to optimize different concentrations of Oleic acid (OA) and Palmitic acid (PA) to be used either alone or in combinations to induce NASH in HepG2 in vitro cell lines. This was tested against oil red staining for fatty acid incorporation, triglyceride content and also the molecular downstream signatures for genes involved in NASH and HCC pathogenesis. After analyzing the results, the most suitable model was chosen to investigate the possible role of Rhm for the treatment of NASH and also HCC models.

## Materials and methods

### Chemicals

All chemicals were of ultra-pure molecular biology grade.

Rhamnetin (17799) Sigma-Aldrich, Oil red O (O0625) Sigma-Aldrich, PA (PO500) Sigma-Aldrich, OA (O1383) Sigma-Aldrich, Bovine serum albumin-BSA (160069), Sigma, USA. Fetal Bovine serum (10270-106), Trypsin-versene (BE17-161E), Trypan blue (17-942E), L-glutamine (17-605E), Antibacterial-Antimycotic (15240062), RPMI Lonza (12-702F), Lonza, USA.

### Cell culture

HepG2 cells were cultured in (RPMI 1640) supplemented with 10% FBS, 1% l-glutamine and 1% (penicillin-streptomycin-amphotericin). Starvation procedure or model applications HepG2 cells were cultured in 5% BSA-RPMI media, 1% L-glutamine and 1% (penicillin-streptomycin-amphotericin), HepG2 cells were incubated at 37 °C and 5% pCO2. At 80% confluency, cells were passaged. Cell viable count was carried out using a hemocytometer as previously described [29].

### Preparation of conjugated PA and OA

As previously described [30, 31], with minor modifications, PA and OA were dissolved in 0.1 M NaOH to prepare sodium salt of PA and OA with a final concentration of 7mM. In addition, BSA in PBS 2 mM was prepared, then conjugation between BSA in PBS and sodium salt of PA and OA provide finalized ratio of 3.5:1 mM (fats: BSA) mixture. Subsequently, this mixture was heated at 70 °C for 30 min and with interrupting vortex till complete dissolution. This conjugated lipid mixture was then left to cool, then filtered for sterilization using a 0.22 µm syringe filter to be ready for use.

### In vitro model induction

The induction protocol lasts for three days. On the first day, cells were cultured in RPMI- BSA 5%, 1% l-glutamine and 1% (penicillin-streptomycin-amphotericin) media, for accommodation with the new space.

After 24 h, media was removed, and the cells were washed by PBS twice. Then different concentrations of PA and OA conjugated with BSA media were added to the well, except for the control where free fats 5% BSA media was used. Different concentrations of conjugated PA and OA were applied to reach the final concentration as follows: OA 100 µM, OA 500 µM, PA 100 µM, PA 500 µM, or combined mixtures of (OA: PA) with final concentration 600 µM at ratios 2:1, 1:1 and 1:2. Also, combined mixtures of (OA: PA) with final concentration 1000 µM at ratios 2:1, 1:1 and 1:2, with overall 10 models not including the control. After 24 h incubation cells were either fixed or collected for staining or biochemical and molecular analysis.

Viable cell count procedure was done before the three days procedure for the insurance of administering equal number of cells per each well in all of the experiments. Cells were counted under the microscope using hemocytometer, then viability was calculated using the following formula

$${\text{Cell}}\,\,{\text{Count}}\, = \,{\text{Number}}\,{\text{of}}\,{\text{counted}}\,{\text{cells}}/\left( {4\, \times \,10^{ - 4} } \right)\, \times \,{\text{initial}}\,{\text{volume}}\, \times \,{\text{dilution}}\,{\text{factor}}$$

### Oil red staining for the fatty acids in the prepared models

For lipid staining, Oil Red stain was purchased from Sigma-Aldrich (O0625). First, the cells were fixed with 10% neutral formalin for 1 h at room temperature. Then the fixed cells were washed with PBS. Subsequently, 60% isopropanol was used for washing and then left for air drying. Filtered OR O was added to each well for 2 h and incubated at 37 °C. Finally, cells were washed with dd.H_2_O and left for air drying for microscopic detection of lipids [32].

### Triglycerides concentration assay

Media was removed from the wells and the cells were washed with PBS twice. Cells were trypsinized and collected as pellets for triglycerides (TG) extraction and quantification. For TG extraction, pellets were resuspended and washed with PBS, then 200 µL methanol and 500 µL chloroform were added to each. Pellets were vortexed and sonicated using a shaker sonicator for 1 h for complete cell disruption and TG extraction. Afterwards, 250 µL of distilled H2O was added, and pellets were vortexed then centrifuged at 5000 rpm for 25 min. The lower phase (organic) was transferred into a new tube and left to air dry overnight [33–35]. Finally, the concentration of TG in each model was determined by the commercially available kits (Spectrum diagnostics, Egypt) following the manufacturer protocol. Briefly, 1 mL reagent was added for 10 min, then the absorbance was measured at 546 nm.

### Determination of the most suitable model for validation against rhamnetin

Ten models were prepared and compared to the control, all models were tested for FFAs incorporation using OR O, where results were all positive and will be presented in the results part. All models were measured for TG concentration, where results showed increase over the control. After the molecular comparison between various prepared models and statistical analysis, the most suitable model to be used for the evaluation of Rhm effect on NASH was PA 500 µM.

### *Determination of IC*_*50*_* for rhamnetin*

The cytotoxicity assay for determination of the IC_50_ concentration of Rhm was done using Vybrant® MTT Cell Proliferation Assay Kit (V-13154) following the manufacturers protocol [24]. After culturing HepG2 cells, adequate cell count was transferred to a 96-well plate with 5000 cells per well. Triplicates for the controls and each concentration of Rhm were planned on the plate. Positive control was HepG2 cells supplemented with DMSO in BSA-media. Then the wells planned for Rhm serial concentration were supplemented with BSA-media and PA 500 µM. The BSA-media was removed from the wells, washed with PBS, and replaced with fresh RPMI-media supplemented with 10 µL of 12 mM MTT stock solution. The plate design included a negative control. The Negative control was prepared by adding 100 µL of fresh medium in a well clear from the cells, and 10 µL of 12 mM MTT stock solution. The plate was incubated at 37 °C for 4 h. Then 100 µL of SDS-HCL solution was added to each well and thoroughly mixed. In a humidified chamber the well plate was incubated at 37 °C for 12 h. Finally, absorbance was measured at 570 nm.

### Quantitative real-time PCR

Extraction of total RNA was done using PureLink™ RNA Mini Kit (12183018A) ThermoScientific co. Revertaid™ ThermoScientific co (K1622) was used for cDNA synthesis, where we used 1 µM/µL RNA template, 1 µL Random Hexamer primer and a total volume of 20 µL based on the manufacturer protocol. Maxima Sybr green kit (K0221) was used for qRT-PCR reactions where the standard kit procedure was performed. StepOne plus instrument was used for qRT-PCR experiment where we used standard provided kit protocol. Relative genetic expression calculations were performed using the 2–ΔΔCt method and normalized to the β-actin gene. Nucleotides sequences of the designed PCR primer pairs for all genes, including GR, NFκB, NFE-2, SREBP-1c, TGF-β, BCL-2, CAT, FAS, IL-6, TNF-and β-actin gene (Promega., USA) are shown in Table 1.

**Table 1:** Sequences of primers used for gene expression analysis

Gene symbol	Primer sequence	Accession No
GR F: GR R:	5′-CGGTGCCAGCTTAGGAATAA-3 5′-TCTCCACAGCAATGTAACCTG-3′	NM_001191002.2
FAS F: FAS R:	5′- AGACACTCGTGGGCTACA-3′ 5′- CCCTCTGAAGTCGAAGAAGAAG-3′	NM_000043.6
NF-κB F: NF-κB R:	5′-GGAAGTACAGGTCCAGGGTATAG-3′ 5′- CCATGCTTCATCCCAGCATTAG-3′	NM_003998.4
NFE-2 F: NFE-2 R:	5′- CCCAGCAGGACATGGATTT-3′ 5′- TGTCATCTACAAACGGGAATGT-3′	NM_001136023.3
BCL-2 F: BCL-2 R:	5′- CCAGTACCTTAAGCCCTGATTG-3′ 5′- CAGAGCCAGTATTGGGAGTTG-3′	NM_000633.3
TGF-β F: TGF-β R:	5′- CAATTCCTGGCGATACCTCAG-3′ 5′- CACAACTCCGGTGACATCAA-3′	NM_000660.7
IL-6 F: IL-6 R:	5′- TCTGGATTCAATGAGGAGACTTG-3′ 5′- CTCTGGCTTGTTCCTCACTAC -3′	NM_000600.5
CAT F: CAT R:	5′- CTCCACTGTTGCTGGAGAAT-3′ 5′- CGAGATCCCAGTTACCATCTTC-3′	NM_001752.4
SREBP F: SREBP R:	5′- GGGACAAGGAATTCTCGGATG-3′ 5′- ATGCTGGAACTGATGGAGAAG-3′	NM_001353623.2
β-actin F: β-actin R:	5′-GGCACCACACCTTCTACAAT-3′ 5′-AACATGATCTGGGTCATCTTCTC-3′	NM_007393.5
TNF-α F: TNF-α R:	5′- CATGTTGTAGCAAACCCTCAAG-3′ 5′- GAAGAGGACCTGGGAGTAGAT-3′	NM_000594.2

*GR* Glutathione reductase; *FAS* Fatty acid synthase; *NF-kB* Nuclear factor-kappa B; *NFE*-2 Nuclear Factor, Erythroid 2; *SREBP*-1c Sterol regulatory element-binding protein; *TGF-β* Transforming growth factor-beta; *IL*-6 Interleukin 6; *BCL*-2 B-cell lymphoma 2; *β-actin* beta-actin; *CAT* Catalase; *TNF- α* Tumor necrosis factor-alpha

### Statistical analysis

Data were expressed as mean ± SEM. Comparison of data was made using analysis of variance (ANOVA) using post hoc test (Tukey's Multiple Comparison Test) tests to compare individual groups in analysis of the TG concentration and the first comparative step of preparing the different NASH models. In case of investigating the effect of Rhamnetin on NASH or HCC unpaired t-test was used because at these two steps we compared two groups. Statistical analyses were conducted using GraphPad prism. A probability of *p*-value < 0.05 is statistically significant.

## Results

### Staining with oil red O showed fatty acid incorporation in cells using models with single or mixed lipids

**Fig. 1:**
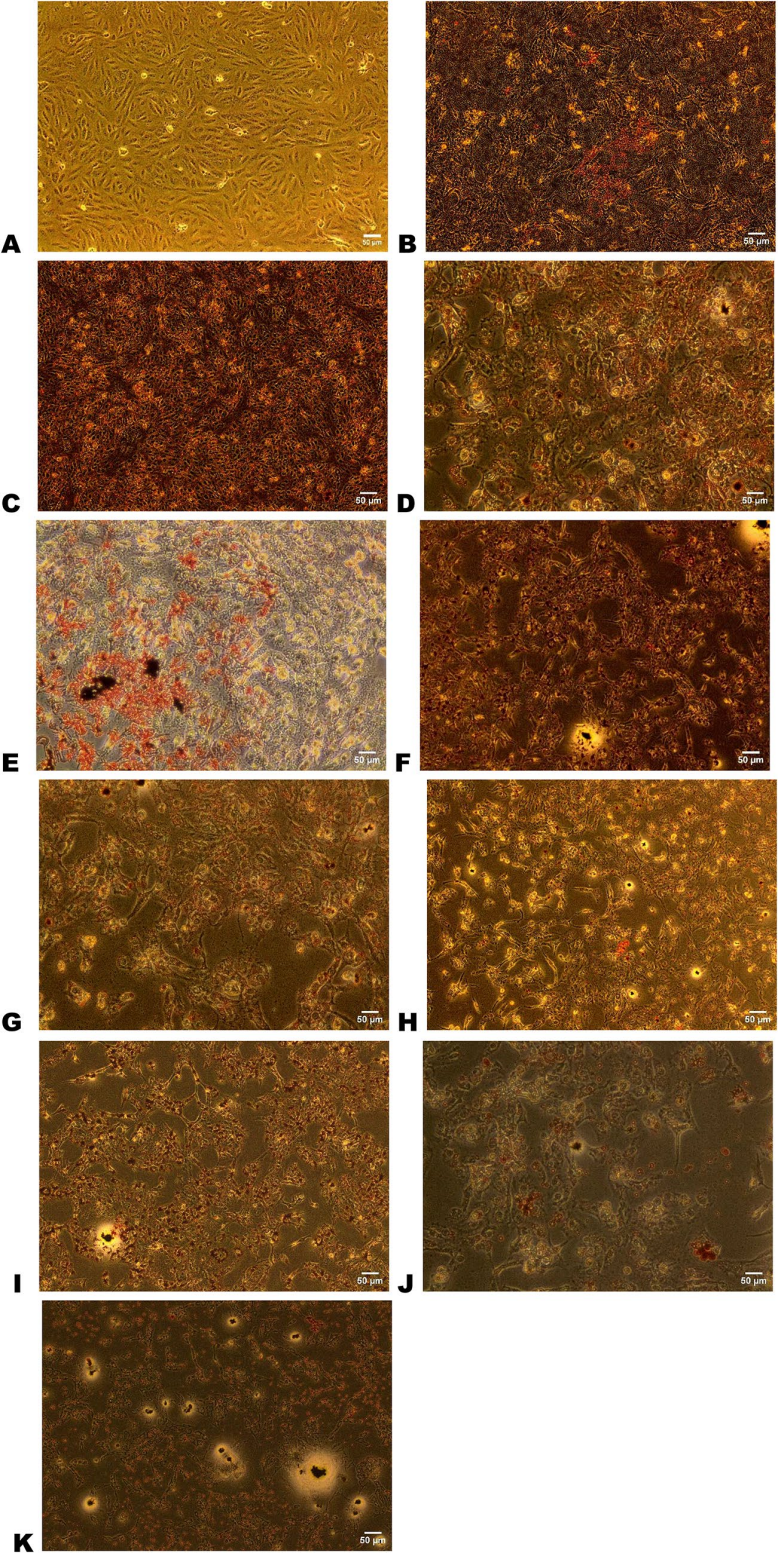
Oil red O staining of fat droplets in different models. This figure represents OR O staining for the models, *n* = 3. Different concentrations of conjugated PA and OA were applied with final concentration as follows: **A** Control, **B** PA 100 µM, **C** PA 500 µM, **D** OA 100 µM, **E** OA 500 µM, **F** mixture of OA:PA of ratio 1:1 and final concentration 600 µM, **G** mixture of OA:PA of ratio 1:1 and final concentration 1000 µM, **H** mixture of OA:PA of ratio 2:1 and final concentration 600 mixture, **I** mixture of OA:PA of ratio 2:1 and final concentration 1000 µM, **J** mixture of OA:PA of ratio 1:2 and final concentration 600 µM, **K** mixture of OA:PA of ratio 1:2 and final concentration 1000 µM

Our results showed that the single addition of PA (100 µM and 500 µM) or OA (100 µM and 500 µM) or the mixed addition of (OA:PA) with final concentration (600 and 1000) µM at ratios 2:1, 1:1 and 1:2 led to accumulation of fatty acids inside cells as indicated by the red lipid droplet formation (Fig 1). All models showed positive staining with OR O compared to control, images were analyzed using ImageJ software as shown in Fig. 1.

### Single and mixed lipid models increased TG content

**Fig. 2:**
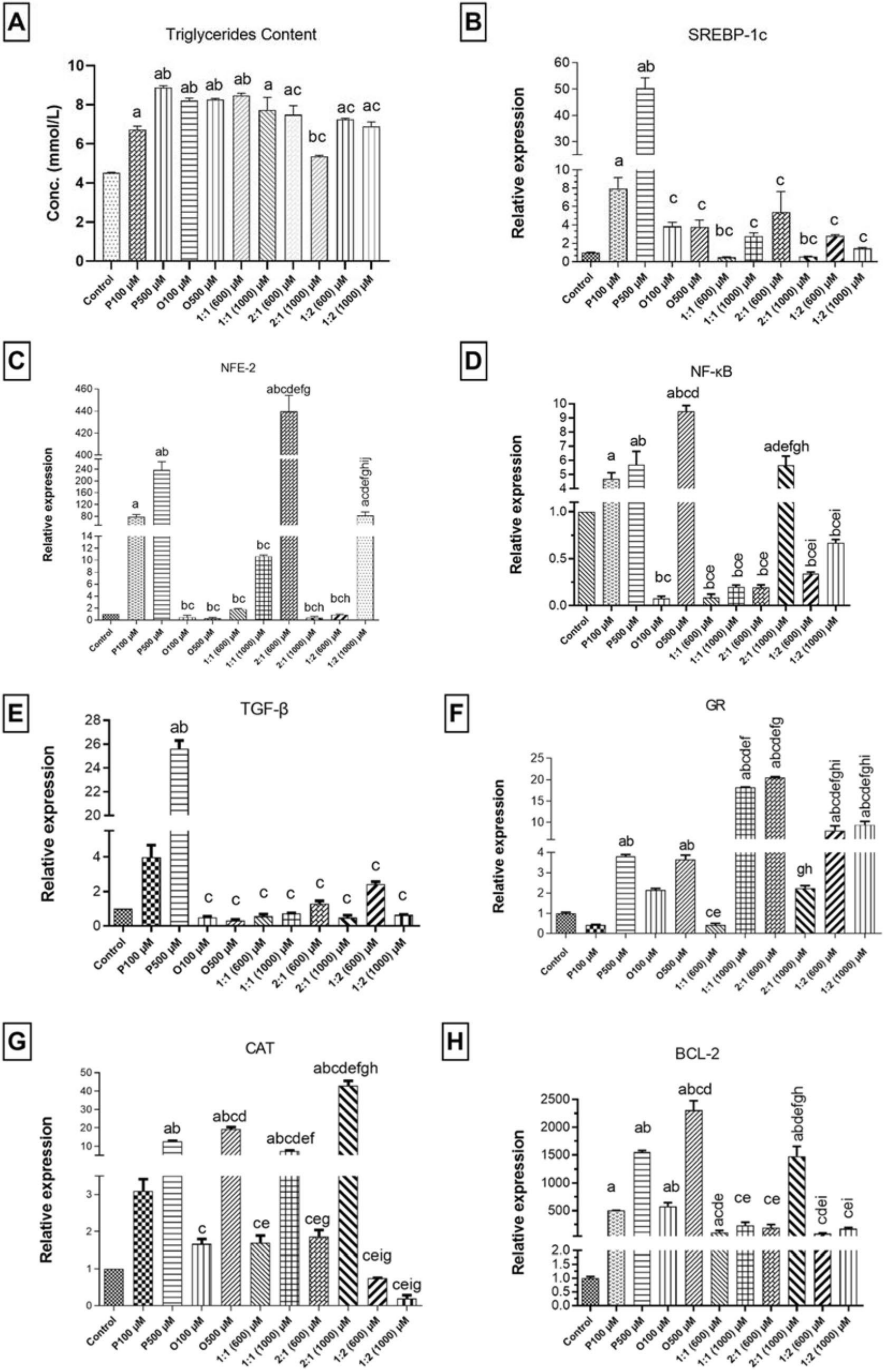
Represents results for the prepared NASH models for triglycerides concentration and the relative expression in the selected genes as follows: **A** Triglycerides concentration, **B** SREBP-1c, **C** NFE-2, **D** NFκB, **E** TGF-β, **F** GR, **G** CAT, **H** BCL-2 in single lipid models and mixture models, where mixture ratios represent OA: PA. All experiments were performed triplicates, *n* = 3. a: Significant difference from control, b: Significant difference from PA 100 µM, c: Significant difference from PA 500 µM, d: Significant difference from OA 100 µM, e: Significant difference from OA 500 µM, f: Significant difference from 1:1 (600) µM, g: Significant difference from 1:1 (1000) µM, h: Significant difference from 2:1 (600) µM, i: Significant difference from 2:1 (1000) µM, j: Significant difference from 1:2 (600) µM, k: Significant difference from 1:2 (1000) µM

Intracellular quantification of TG showed significant results in all models. Where TG concentration was found to be statistically significant in all models except for OA:PA of ratio 2:1 and final concentration 1000 µM where the increase was not significant. The increased percentages for TG content in all other remaining models were higher than those found in case of control cells as follows 48.94%, 96.52%, 81.70%, 83.06%, 87.74%, 70.90%, 65.78%, 18.58%, 60.29%, and 52.60%, in PA 100 µM, PA 500 µM, OA 100 µM, OA 500  , mixture of OA:PA of ratio 1:1 and final concentration 600 µM, mixture of OA:PA of ratio 1:1 and final concentration 1000 µM, mixture of OA:PA of ratio 2:1 and final concentration 600 µM, mixture of OA: PA of ratio 2:1 and final concentration 1000 µM, mixture of OA:PA of ratio 1:2 and final concentration 600 µM, and mixture of OA:PA of ratio 1:2 and final concentration 1000 µM, respectively. The highest increase was observed in case of PA 500 µM concentration, as shown in Fig. 2.

### RT-qPCR results in NASH comparative step

*De-novo lipogenesis* was assessed by determining the expression of SREBP-1c level as a major regulator in this pathway [36, 37]. Results showed significant increase in SREBP-1c relative expression with a *p*-value <0.0001. Only PA 100 µM and PA 500 µM were statistically significant and increased by eightfold and 50 folds, respectively. The highest SREBP-1c relative expression was associated with PA 500 µM. SREBP-1c increased, with similar level, in both OA 100 µM and OA 500 µM about 3.8 folds the control. Besides, PA 500 µM showed significant relative increase as compared to other models where it increased above PA 100 µM, OA 100 µM, OA 500, mixture of OA:PA of ratio 1:1 at final concentrations of 600 µM, mixture of OA:PA of ratio of 1:1 and final concentration 1000 µM, mixture of OA:PA of ratio 2:1 and final concentrations 600 µM, mixture of OA:PA of ratio 2:1 and final concentrations 1000 µM, mixture of OA:PA of ratio 1:2 and final concentrations 600 µM, and mixture of OA: PA of ratio 1:2 1000 µM by 6666, 10, 13, 100, 18, 9, 87, 17, and 34 folds, respectively, as shown in Fig. 2.

*Inflammation* is another major hallmark of NASH. Our results showed variability in related markers in most of our models compared to the control, where the tested genes were NFE-2 [38], NFκB [4] and TGF-β. The latter is also considered a fibrotic marker as well [39, 40]. All those markers showed statistical significance with *p*-value <0.0001 as compared to the control.

Firstly, NFE-2 showed statistically significant relative expression increase in PA 100 µM, PA 500 µM, mixture of OA:PA of ratio 2:1 and final concentration of 600 µM, and mixture of OA:PA of ratio 1:2 and final concentration of 1000 µM by 78, 237, 439 and 82 folds, respectively. Contrary effect between PA and OA single lipid models occurred, where PA 100 µM and PA 500 µM were upregulated, while OA 100 µM and OA 500 µM models were downregulated. Besides PA 500 µM showed statistically significant result compared to all other models.

Secondly, NFκB showed significant relative expression increase in PA 100 µM, PA 500 µM, OA 500 µM, and mixture of OA:PA of ratio 2:1 and final concentration 1000 µM, which were increased by 4.7, 5.7, 10.8, and 5 folds, respectively. The highest relative expression appeared in OA 500 µM followed by PA 500 µM; both models showed a significant relative expression increase compared to all other models. Finally, OA 100 µM, opposite to OA 500 µM, showed downregulation.

Thirdly, TGF-β results only showed a significant relative expression increase in PA 500 µM by 25.6 folds, as well as being significant with the other models, as shown in Fig. 2.

*Oxidative stress* was assessed by determining the relative expression of GR and CAT expressions [41]. Results showed significance with a *p*-value <0.0001 in both genes. In GR results showed significant relative expression increase in PA 500 µM, OA 500 µM, mixture of OA:PA of ratio 1:1 and final concentration 1000 µM, mixture of OA:PA of ratio 2:1 and final concentration 600 µM, mixture of OA:PA of ratio 1:2 and final concentration of 600 µM, and mixture of OA:PA of ratio 1:2 and final concentration 1000 µM by 3.8, 3.65, 18.18, 20.47, 8.06, and 9.45 folds, respectively. The highest relative expression increase appeared in the mixture of OA:PA of ratio 2:1 and final concentration of 600 µM, while a decrease in the relative expression was found in both PA 100 µM and mixture OA:PA of ratio 1:1 and final concentration 600 µM. In GR, the mixture models were generally increased above single lipid models. Besides, PA 500 µM had a statistically significant difference from all of the mixture models except mixture of OA:PA of ratio 2:1 and final concentration 1000 µM, as shown in Fig. 2.

Moreover, in CAT results showed significant relative expression increase in PA 500 µM, OA 500 µM, mixture of OA:PA of ratio 1:1 and final concentration 1000 µM, and mixture of OA:PA of ratio 2:1 and final concentration 1000 µM by 12.65, 19.39, 7.26, and 42.93 folds, respectively. CAT highest relative expression increase occurred in the mixture of OA:PA of ratio 2:1 and final concentration of 1000 µM. On the contrary, the mixture of OA:PA of ratio 1:2 and final concentration of 600 µM and the mixture of OA:PA of ratio 1:2 and final concentration of 1000 µM showed significant relative expression decrease upon comparison with the control.

In CAT PA 500 µM was statistically significant with all other models although it was not the highest relative expression result, as shown in Fig. 2.

*Apoptosis* is considered a significant marker for NAFLD progression [42]. Results showed a significant relative expression increase with a *p*-value <0.0001 in the anti-apoptotic BCL-2 expression. BCL-2 results significant relative expression increase in PA 500 µM, OA 100 µM, OA 500 µM and mixture of OA:PA of ratio 2:1 and final concentration 1000 µM by 1500, 575, 2307, and 1473 folds, respectively. BCL-2 highest significant relative expression increase was presented by OA 500 µM, followed by PA 500 µM. Generally, all models showed an increase in relative expression compared to the control, the high relative expression results may be due to high stress affecting the cells. Again PA 500 µM showed statistical significance compared to all models except for mixture of OA:PA of ratio 1:1 and final concentration 600 µM, as shown in Fig. 2.

Here we constructed a table summarizing the different genetic molecular results emerging from the preliminary experiments to illustrate the effect of FAs induced NASH models on the molecular level using OA, PA, or a mixture of both. This table showed the mean relative expression in the models in comparison with the controls whether upregulation or downregulation occurred. Then statistical significance of these relative expressions was illustrated in each gene for each model. Upon deep comparison of the results, we found that PA 500 µM model showed statistically significant results in all genetic hallmarks related to NASH; thus, it is the most suitable model to investigate the effect of Rhm on FA induced NASH model molecularly in this study but may not be the perfect choice with other NASH inducers or drugs. This method can be used for wider experimentations using different concentrations, or ratios, or other inducing factors; thus, aiding in identification of inducers as effecters and results as affecters, and determination of the suitable model for investigation of targeted drugs, as shown in table 2.

**Table 2:** Represents results of gene expression of various markers used in the comparison between NASH induced models in order to choose the most suitable model

Models/Genes	De-novo lipogenesis	Inflammation			Oxidative stress		Apoptosis
	SREBP-1c	NFE-2	NFκB	TGF-β (fibrosis)	GR	CAT	BCL-2
Control							
PA 100 µM	7.95*	78***	4.7*	*3.96*	*0.42*	*3.08*	503.25*
PA 500 µM	50.27***	237.37***	5.70***	20.94***	3.8**	12.65***	1548.85***
OA 100 µM	*3.84*	*0.5*	*0.07*	*0.49*	*2.14*	*1.66*	575.85**
OA 500 µM	3.76	*0.31*	9.4***	*0.31*	3.65**	19.39***	2307.91***
1:1 (600) µM	*0.5*	*1.82*	*0.08*	*0.55*	*0.42*	*1.69*	*108.27*
1:1 (1000) µM	*2.77*	*10.65*	*0.19*	*0.71*	18.18***	7.26***	*228.26*
2:1 (600) µM	*5.39*	439.75***	*0.19*	*1.29*	20.47***	*1.86*	*189.27*
2:1 (1000) µM	*0.57*	*0.43*	5.66**	*0.48*	*2.24*	42.93***	1473.26***
1:2 (600) µM	*2.82*	*0.95*	*0.33*	*2.42*	8.06***	*0.74*	*85.16*
1:2 (1000) µM	*1.45*	82***	*0.67*	*0.63*	9.45***	*0.19*	*173.64*

Summary of the relative expression results of each gene in the induced NASH models

The bold indicates statistically significant results, and italics indicates non-significant results

*n* = 3

*Significantly changed from the control at *p* < 0.05. **Significantly changed from the control at *p* < 0.01. ***Significantly changed from the control at *p* < 0.001

### *Determination of the IC*_*50*_* of rhamnetin*

**Fig. 3:**
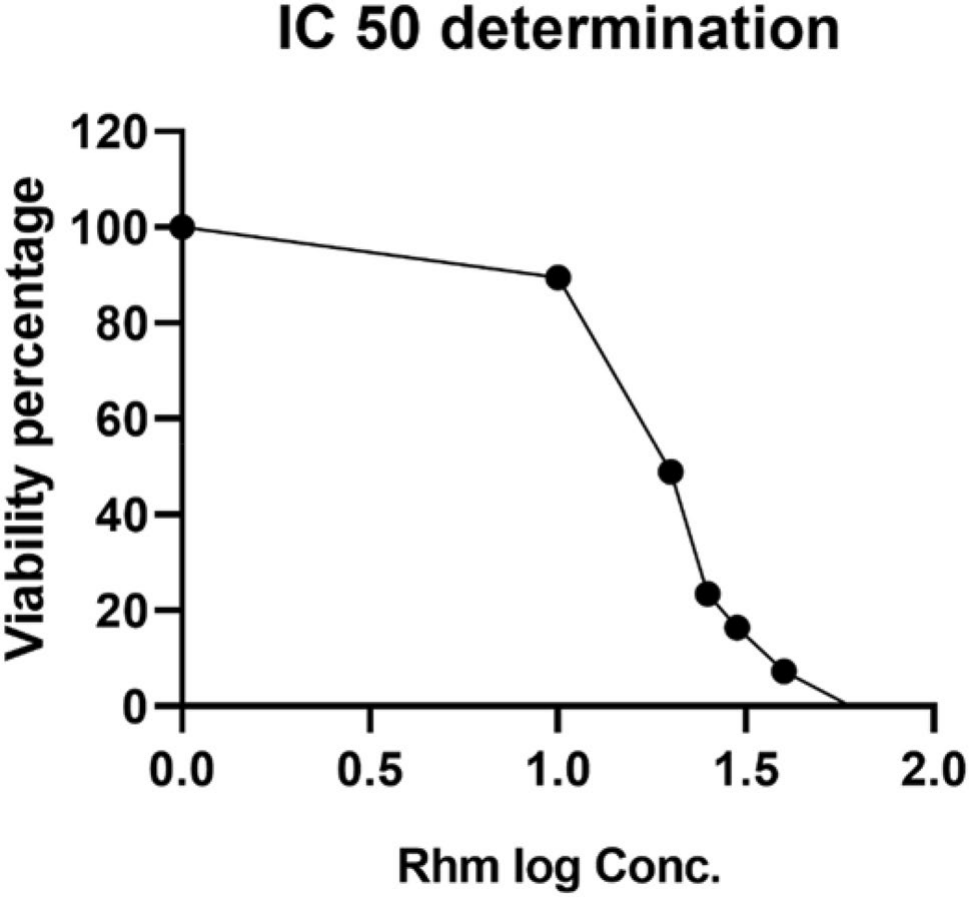
Figure illustrates IC_50_ determination which shows the results of the MTT assay to determine the IC_50_ concentration for Rhamnetin; Rhm log Conc.: Log concentration of Rhamnetin concentrations used; viability: normalized absorbance value detected during the experiment showing highest viability percentage instead of highest absorbance value and lowest viability percentage with lowest absorbance value

The cytotoxicity assay results were extracted, then GraphPad Prism was used for calculating the IC_50_ which was used for further experimentation. Results showed that Rhm cytotoxicity level showed log IC_50_ = 0.1065, and IC_50_ = 1.278 as shown in Fig. 3. These results provide that the targeted concentration to be used is 18.967 µM, that was approximated to 19 µM for more practical performance.

### RT-qPCR results for the investigation of rhamnetin effect on PA 500 µM induced NASH model

**Fig. 4:**
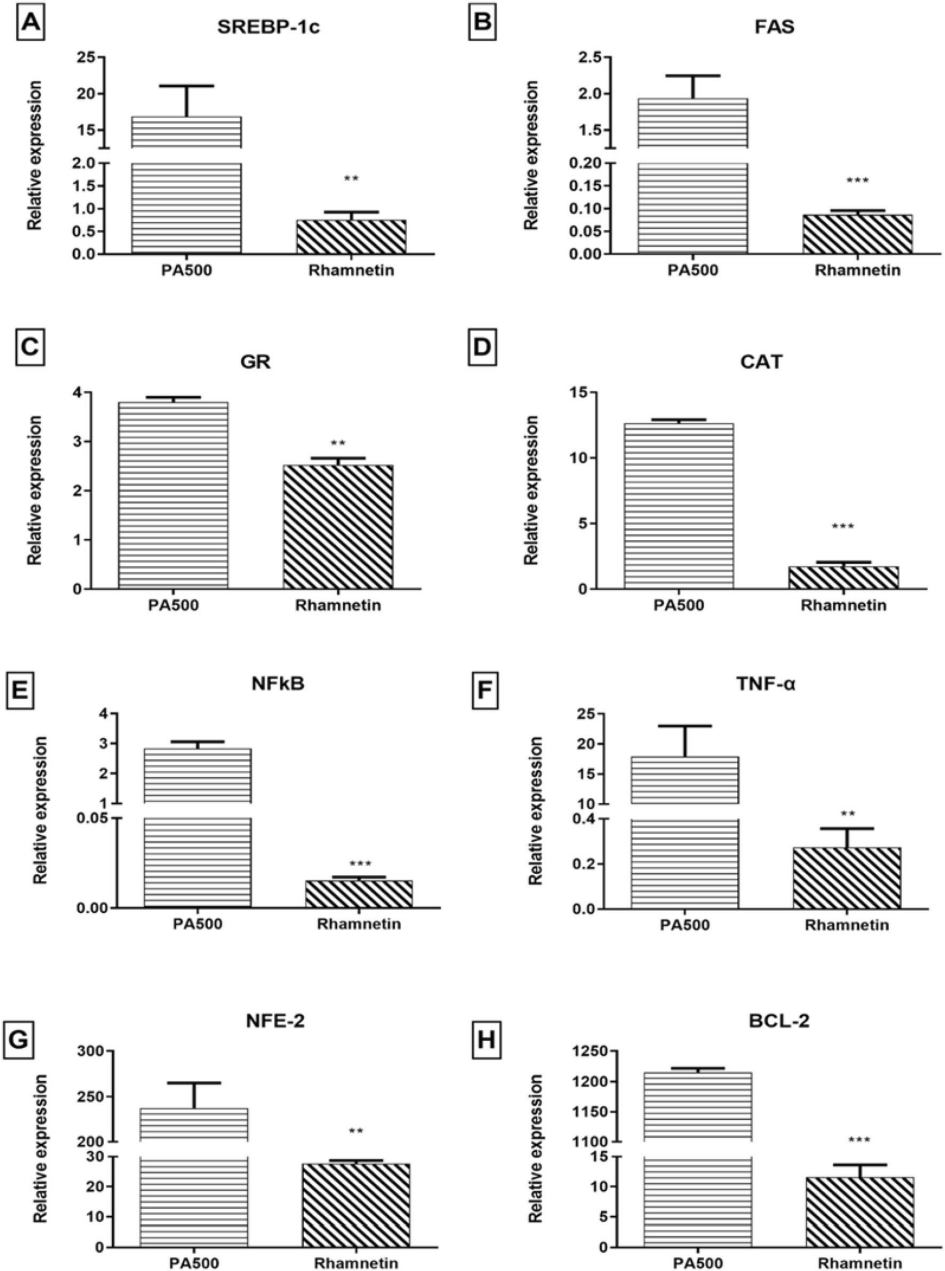
Shows the effect of Rhamnetin on gene relative expression in NASH hallmarks in PA 500 µM induced model. Selected genes included: **A** SREBP-1c, **B** FAS, **C** GR, **D** CAT, **E** NFκB, **F** TNF-α, **G** NFE-2, **H** BCL-2. *n* = 3. *Significantly changed from the control at *p* < 0.05, **Significantly changed from the control at *p* < 0.01, ***Significantly changed from the control at *p* < 0.001; PA 500: NASH model induced using PA with concentration of 500 µM, Rhamnetin: NASH model induced using PA with concentration of 500 µM and treated with Rhamnetin for investigating the effect of Rhamnetin on NASH

Rhamnetin was assessed to ameliorate NASH in vitro model induced using PA 500 µM as it was the most suitable model based on the previously mentioned results. Rhm’s effect was tested on several genes including SREBP-1c and FAS for the de-novo lipogenesis, GR, and CAT for the oxidative stress, NFκB, TNF-α, and NFE-2 for the inflammatory machinery, and finally BCL-2 for apoptosis. The results showed ameliorative effect proving that Rhm is effective in treatment of FAs induced NASH. Results showed significant relative expression decrease in all of the aforementioned genes with p-values of 0.0086, 0.001, 0.0015, <0.0001, <0.0001, 0.0087, 0.0017, <0.0001 for SREBP-1c, FAS, GR, CAT, NFKB, TNF-α, NFE-2, and BCL-2, respectively, as shown in Fig. 4.

### RT-qPCR results for the investigation of rhamnetin effect on HCC

**Fig. 5:**
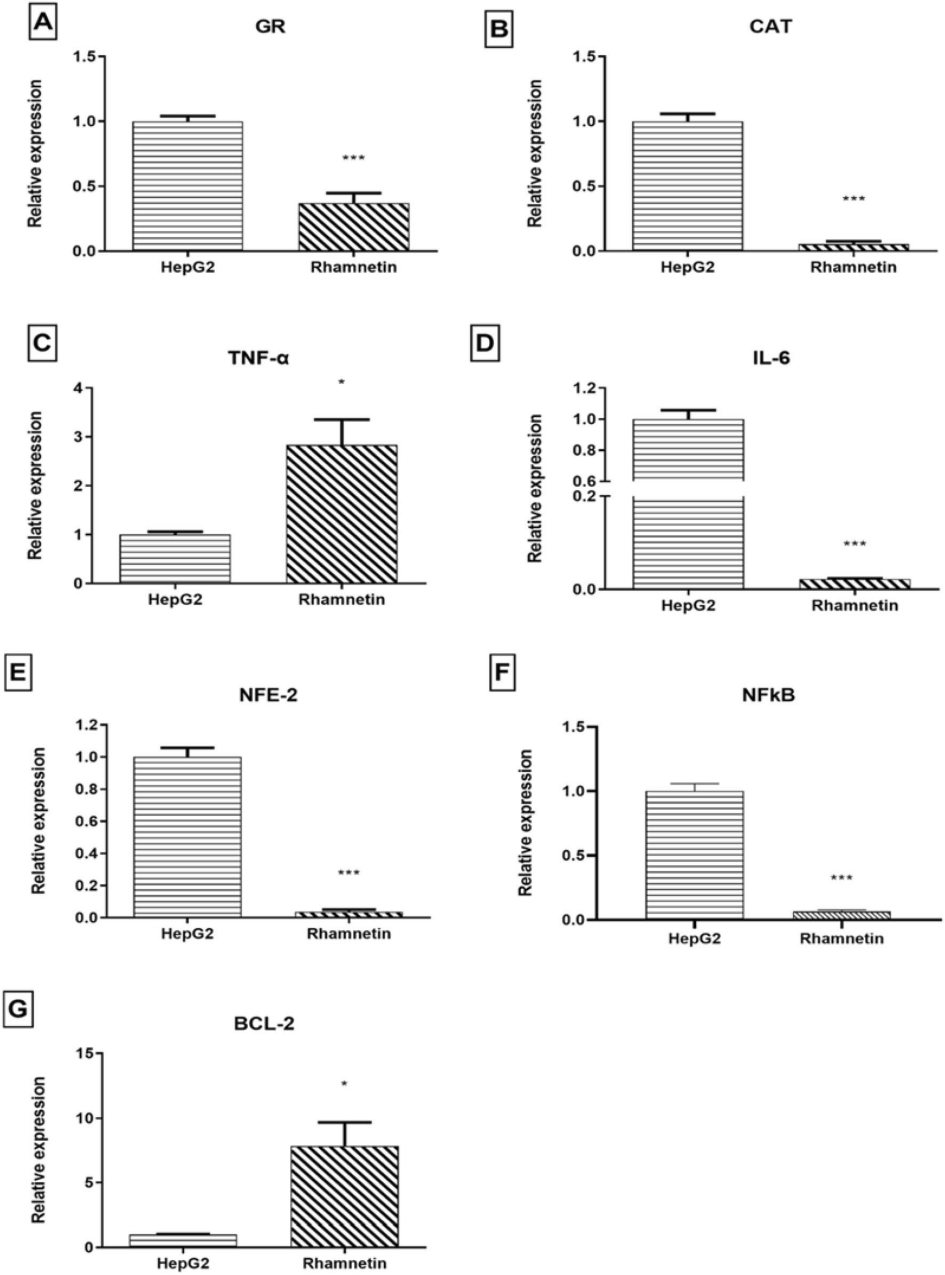
Shows the effect of Rhamnetin on gene expression on HCC hallmarks expressed in HepG2 Cells. Selected genes included: **A** GR, **B** CAT, **C** TNF-α, **D** IL-6, **E** NFE-2, **F** NFκB, **G** BCL-2. *n* = 3. *Significantly changed from the control at *p* < 0.05, **Significantly changed from the control at *p* < 0.01, ***Significantly changed from the control at *p* < 0.001; *HepG*2 HepG2 cells representing HCC control, Rhamnetin: HepG2 cells supplemented with Rhamnetin to detect the effect of Rhamnetin on HCC

Rhamnetin effect on HCC was investigated on some of the previously mentioned hallmarks. Results showed statistically significant relative expression decrease in GR and CAT, with *p*-value = 0.0004, and 0.0001 respectively. While the inflammatory machinery showed statistically significant relative expression decrease in NFκB, NFE-2, and IL-6, with *p*-value < 0.0001 for the 3 genes. But the anti-inflammatory effect did not affect TNF-α in addition to being upregulated. Finally, Rhm effect on apoptosis was not significant where BCL-2 was upregulated as shown in Fig. 5.

## Discussion

Metabolic syndromes are still hard to control. NAFLD is one of those metabolic syndromes as it is associated with obesity, T2DM [43], dyslipidemia, and heart disorders [44]. In this study, Rhamnetin (Rhm) was proven to improve NASH status *in vitro* and improved the molecular map in HCC and this may be a promising therapeutic agent for decreasing NAFLD progression. In addition, the use of any NASH inducer to investigate the effect of a promising drug should be perfectly optimized as the molecular expression differs according to the kind, ratio, and concentration of the inducer used, For example FAs affects NASH stage and consequently produce different genetic expression in each case. Any drug of choice may have an expected target, therefore, the model chosen for the experiment must show relevant molecular expression, and otherwise several models must be compared to determine an optimum condition for every specific research. Finally, drugs used for NAFLD treatment having multiple effects as the case in Rhm therapeutic modality which can aid in ameliorating different progressive stages and therefore might have better impact in treatment. As well as, choosing a suitable model for the experiment will be easier due to the presence of several targets to measure.

The lack of an FDA approved drug for this disease keeps the door open for extensive studies. Lack of therapeutic strategies justifies the importance of preliminary studies for high throughput screening. Herein, we constructed different models that mimic NAFLD/NASH with downstream molecular investigations that help in providing a platform for high-throughput screening of various potential drugs. NAFLD pathogenesis theory is now chiefly based on the multiple-hit progression which includes the interplay between oxidative stress, inflammatory, de-novo lipogenesis, and apoptotic machinery key players.

The construction of a cellular model that mimics liver steatosis in vitro could facilitate screening of various potential drugs and allow studying the molecular progressive pathways of the disease associated with the development of HCC [16, 45]. First, we established several possible models, then we validated steatosis using OR O staining and measured the TG concentration, finally, we determined the change in the level of genetic relative expression. Several studies had relied on HepG2, an available, affordable, and reliable choice that can be used in reproducible studies [46, 47] In vitro studies have drawbacks, such as the inability to express human body tissue complexity. However, in vitro experiments are developed quickly and had fewer ethical regulations, making them the preferable approach for screening and preliminary studies. Finally, in vitro studies allowed us to compare several concentrations and mixtures quicker where cells have sustained growth and reproducibility with relatively similar results [48, 49]. The target was to reach steatotic models mimicking NAFLD/NASH. After we confirmed models success by OR O staining and TG concentration, the significant change in relative expression that matched the KEGG platform for NAFLD presented significant explanation for the genetic map, that matched previous reports [17–19, 32, 36]. OR O staining showed a clear presentation of TG incorporation in the prepared models, upon comparison to the control. Density of the stain is reflecting the amount of lipids incorporated, most models where roughly similar with minor variations that may be related to kind and amount of fats used to prepare each model. The successful lipid incorporation allows fundamental usage of any prepared model in this study. Next investigating the TG concentration in each model generally showed high level compared to the control, but not necessarily a dose-dependent increase. The increase in TG concentration comes as a second fundamental approval for usage of any model in this study. The first and second fundamental outcomes are of importance in progressing to the next stage of investigating the difference in relative expression of the molecular targets in these models.

In a clinical study, liver biopsies were taken from patients with NAFLD/NASH of different degrees of the disease and analyzed for the abundance of different FAs. PA and OA were found to be from the six increasing FAs in serum of NASH patients [50]. Earlier, other studies proved that NAFLD/NASH is associated with dysregulation in plasma circulating lipidome [51]. Some studies had chosen OA and PA to initiate steatosis based on the fact of being relevant to fat accumulation in the liver [7]. Our study outlined that FAs effect in a mixture differs from their effect when added separately to induce NASH in vitro. Whereas the effect of PA or OA when added separately was associated with the highest relative expression of genetic differences than their effect if added in combination. Herein, the resulting effect of combining OA and PA causes cell death and reprogramming of oxidative stress [52]. We found that PA alone, exceptionally the high concentration of 500 µM induced cytotoxicity concomitantly with previous studies [53, 54].

Therefore, PA 500 µM model was compatible with the expected results of NAFLD/NASH molecular map (https://www.genome.jp/pathway/map04932). Also PA as a saturated fatty acid was reported to increase lipid accumulation induced apoptosis, causing increase in mitochondrial apoptotic pathway, Thus, progressing NAFLD to NASH [55]. In this context, previous studies showed that the effect of PA is more harmful compared to OA in the increase of endoplasmic reticulum (ER) stress, while OA does not affect ER status [52]. Unsaturated fatty acids may be less toxic since they can be esterified. While, saturated fatty acids are believed to increase ER stress, which results in increased reactive oxygen species (ROS) production and induces several inflammatory and apoptotic cascades [56]. Other studies stated that mono-unsaturated FA as OA is less toxic than saturated FA as PA. Besides, PA deteriorates NADH/NAD+ ratio and disconnects glycolysis with TCA, increasing ROS radicals [57]. Thus, OA decreases the cytotoxicity induced by PA, and this may be due to the decrease of oxidative stress due to the fact that OA is an unsaturated FA [58]. This agrees with our results which showed that the effect of mixtures on the molecular genes expression is much lower than the effect of simple lipids. The choice of FAs is accommodating with the new trend of non-invasive diagnosis for NASH that depends on metabolic components, that aid in dominating of personalized medicine [59].

Usage of multi-factorial genetic targets was emerged from the fact that NASH is a multifactorial disease, as well as it was found to have several categories and stages. Proofs showed decrease in the steatotic level with improvement in the liver histology, and another case did not show deterioration in fibrosis although steatosis was increased, thus, NAFLD has not a clear cut pathophysiology [60]. The difference in genetic expression in each model denies any fundamental acceptance of using any model in any case, as each model showed different genetic map, Thus, the choice of a useful model depends on the target of each study. Not mentioning that in case of inducing NAFLD/NASH model with other inducers than OA or PA, mostly the genetic expression will differ.

Lipid accumulation is critical in triggering inflammation showing disease complexity [61]. SREBP-1c expression was supported by increasing lipid accumulation, primarily due to peroxidation [62]. Besides, it induces fatty acid biosynthesis and plays a crucial role in developing NAFLD [63]. In alignment, our results showed that PA 100 µM and 500 µM showed increased significance in SREBP-1c gene expression as compared to the control.

The development of steatohepatitis inflammation is primarily affected by NFκB as it is highly expressed in early stage of NAFLD/NASH [64]. Furthermore, inflammatory mediators as NFE-2 and TGF-β were found to be both significantly increased in PA 500 µM, while NFE-2 was found significantly increased alone in PA 100 µM, mixtures of OA:PA of ratio 2:1 with final concentration of 600 µM and mixture of OA:PA of ratio 1:2 with final concentration of 1000 µM which aligns with previous reports [65, 66]. Knowing that TGF-β expression is associated with liver fibrosis, which is a significant risk factor for the disease progression to NASH or even HCC [67]. Noted that our results showed that PA 500 µM showed significance in TGF-β expression.

On the other side, GR and CAT were selected to assess oxidative stress state [41, 68]. Cellular redox status deterioration is a hallmark in NAFLD/NASH that affects enzymatic activity [62]. The increase of oxidative stress markers is considered as protective reflex mechanism due to stress on the cells [69, 70]. Similarly, anti-apoptotic BCL-2 was found to be significantly increased in several models. This study showed that PA 500 µM was the only consistent model in applying the expected molecular map for NAFLD/NASH. Furthermore, PA 500 µM was the most reliable and suitable model to mimic anticipated results based on KEGG analysis for NASH. Results make this model a highly recommended one for investigating a potential drug. Merely PA 100 µM is similar to PA 500 µM but with lower relative expression in most genes, however, the statistical significance of the results was in favor of the higher concentration.

We found that the mixtures had a higher relative expression level in GR than single lipid models on the oxidative stress side. Mixtures of OA: PA of ratio 1:1 and a final concentration of 1000 µM and mixture of ratio 2:1 of a final concentration of 600 µM had the highest levels of relative expression, While on the contrary in case of CAT, single lipid models had higher relative expression than mixture models, except with mixture model of OA: PA of ratio 2:1 with final concentration of 1000 µM. OA 500 µM had higher relative expression than PA 500 µM in GR, stating that OA had oxidative stress more than PA. Whereas CAT importance is related to the progression to HCC [71]. For the mixture models, anti-apoptotic genes were found to be significantly increased in the case of OA: PA of ratio 2:1 and the final concentration 1000 µM that had the highest relative expression was found in 2:1 (1000) among the mixtures. Which clarifies the possible protective effects due to the presence of OA, which agrees with the results of Xing. et al. [52]. At the end of the preliminary stage, results of the relative molecular expression of the molecular targets in each model showed wide variation. As a conclusion, the different models induced were not all suitable for this study even after showing positive results in OR O and TG concentration assays. The variation between models may be occurred due to developing different stages of NASH, and different stages occur due to the difference in the stress occurring by the model inducer. Furthermore, statistically significant results in each model is considered incomplete method of judgement on the expression of such gene in models because the correlation of any gene to NASH is not a must that this gene is typically present in any NASH induced model. Therefore, using the multiple comparisons of means in each model is more accurate in evaluating experimental models.

Rhm is a polyphenolic compound that reversed PA 500 µM induced NASH toxicity and showed promising effect by modulating targeted genes. The drug of choice here reversed oxidative stress resulted by decreasing the relative expression of both GR and CAT. Furthermore, anti-progressive effect was clear by decreasing de-novo lipogenesis cascade by suppressing SREBP-1c and FAS, as de-novo lipogenesis is one major NASH hallmark that is one of the major causes of hepatic steatosis. The effect of Rhm on inflammatory makers was significant as well, where NFκB was suppressed. Rhm suppression of inflammatory markers such as NFκB, a promotor of major inflammatory cascade in NASH [72], was reported before [22], but in this study it was reported in NASH model and HCC. The suppression effect of Rhm on BCL-2 was hard to explain whether this effect was protective or toxic to the cells, however, it was reported that steatosis is associated with apoptotic effect clinically in NASH patients [72]. Thus, more studies are required to investigate the effect of apoptosis on inflammation in NASH and specifically BAX levels are of high importance.

In case of HCC, Rhm showed decrease in the oxidative stress that is found originally in cancerous cells as proven by downregulating GR and CAT. Moreover, it showed essential anti-inflammatory effects by suppressing NFκB, IL-6, and NFE-2. However, Rhm activity did not reverse TNF-α and this may be due to the anticancer activity of Rhm, but it was contradicted with increase in anti-apoptotic BCL-2.

Rhm as a potent flavonoid that has numerous activities reported was chosen for this validation to investigate its effect on both NASH and its common complication HCC [73]. Rhm various activities aided in the selection of various genetic targets found in NASH pathophysiology as Rhm pharmacology is still not fully studied [74]. Toxic effect of FAs had been extensively ameliorated using Rhm, this effect may be due to the recessive effect of FAs, or the potency of Rhm, thus, further in vivo experimentation is needed.

Cancerous cell lines offer unlimited growth and stable phenotype, streamline standardized culture protocols and assay reproducibility. Primary non-cancerous cells have limitation in culturing time, availability as well as heterogeneity especially for healthy controls. HepG2 is a cancerous cell line that is widely used for liver research.

It is important to mention that this is a preliminary study that shows primary steps of NASH research that is recommended to be followed by other researchers. Deeper studies including *in vivo* and clinical studies is a must for further clarifications of those results. More investigations including protein assays are needed for confirmation of results, however, it is preferred to be done on animal models to take in consideration tissue environment effects like cells’ variability. NASH is a complex disease affecting humans, all the progressive factors that trigger NASH in humans cannot all be fully simulated using in vitro model*.* Otherwise, factors used for the experiments will be of infinite probabilities. Experimental limitations could be illustrated in the correct administration of BSA as a carrier for FAs, as the final concentration must be calculated by addition of concentrations of both the FA-BSA mixture, and the media used for completion of the final volume. Dependence on OR O for confirmation of fatty acids incorporation in the cells is critical yet considered only a qualitative step that must be followed by triglycerides concentration assays. Rhm was not previously investigated against HepG2 cells using the conditions implemented in this study, consequently toxic effects on the cells are not taken into consideration. Although HepG2 cells were used previously in NASH studies [75, 76] progression and fibrotic levels are not practically obvious in vitro as the study is based on a cancerous cell line rather than a tissue.

In conclusion, herein we describe a new promising approach to minimize research hastle, diagnosis uncertainty and staging probability by providing a platform for drug screening and determining the expression level of molecular markers on several checkpoints. PA 500 µM showed a correct and typical model in impacting the investigated hallmarks by increasing the molecular targets in this study. Finally, this study has established an in vitro hepatic steatosis model and compared with deep and downstream molecular investigations and cellular staining and imaging. We believe that this could facilitate screening of various potential drugs for NAFLD treatment in the near future. Furthermore, we validated this preliminary study with Rhm which is a potent flavonoid that ameliorated the induced NAFLD/NASH model and also HCC.

## Data Availability

All data generated or analyzed during this study are included in this published article.

## Abbreviations

NAFLD: Non-alcoholic fatty liver disease
NASH: Non-alcoholic steatohepatitis
HCC: Hepatocellular carcinoma
PA: Palmitic acid
OA: Oleic acid
OR O: Oil red O stain
PBS: Phosphate buffer saline
GR: Glutathione reductase
NFκB: Nuclear factor kappa B
NFE-2: Nuclear factor erythroid 2
SREBP-1c: Sterol regulatory element-binding protein-1c
FAS: Fatty acid synthase
IL-6: Interleukin 6
TNF-α: Tumor necrosis factor-beta
TGF-β: Transforming growth factor-beta
CAT: Catalase
Bcl-2: B-cell lymphoma 2
β-actin: Beta-actin
FDA: Food and drug administration
KEGG: Kyoto encyclopedia of genes and genomes
